# 2-(3,4,5-Trimethoxy­phen­yl)-1*H*-pyrrolo[2,3-*b*]pyridine

**DOI:** 10.1107/S1600536809047540

**Published:** 2009-11-14

**Authors:** Roland Selig, Dieter Schollmeyer, Wolfgang Albrecht, Stefan Laufer

**Affiliations:** a Eberhard-Karls-University Tuebingen, Auf der Morgenstelle 8, 72076 Tuebingen, Germany; b University Mainz, Duesbergweg 10-14, 55099 Mainz, Germany; c c-a-i-r biosciences GmbH, Paul-Ehrlich-Strasse 15, 72076 Tuebingen, Germany

## Abstract

In the title compound, C_16_H_16_N_2_O_3_, the 3,4,5-trimethoxy­phenyl group makes a dihedral angle of 10.04 (7)° toward the 1*H*-pyrrolo[2,3-*b*]pyridine system. The crystal structure displays inter­molecular N—H⋯N hydrogen bonds, forming inversion dimers.

## Related literature

For the synthesis of the title copmpound, see: Davis *et al.* (1992 ▶)
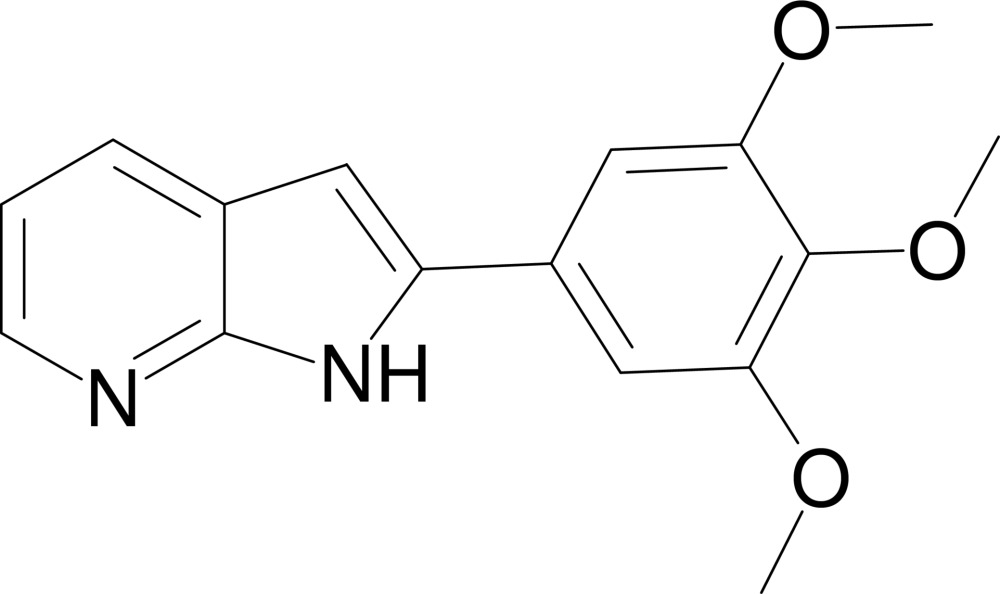


## Experimental

### 

#### Crystal data

C_16_H_16_N_2_O_3_
*M* *_r_* = 284.31
Monoclinic, 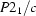

*a* = 7.6283 (9) Å
*b* = 10.1745 (4) Å
*c* = 18.604 (2) Å
β = 104.778 (6)°
*V* = 1396.2 (2) Å^3^
*Z* = 4
Cu *K*α radiation
μ = 0.78 mm^−1^
*T* = 193 K
0.40 × 0.40 × 0.25 mm

#### Data collection

Enraf–Nonius CAD-4 diffractometer
Absorption correction: none
2865 measured reflections
2657 independent reflections
2352 reflections with *I* > 2σ(*I*)
*R*_int_ = 0.020
3 standard reflections frequency: 60 min intensity decay: 2%

#### Refinement

*R*[*F* ^2^ > 2σ(*F* ^2^)] = 0.045
*wR*(*F* ^2^) = 0.125
*S* = 1.07
2657 reflections
193 parameters
H-atom parameters constrained
Δρ_max_ = 0.20 e Å^−3^
Δρ_min_ = −0.21 e Å^−3^

### 

Data collection: *CAD-4 Software* (Enraf–Nonius, 1989 ▶); cell refinement: *CAD-4 Software*; data reduction: *CORINC* (Dräger & Gattow, 1971 ▶); program(s) used to solve structure: *SIR97* (Altomare *et al.*, 1999 ▶); program(s) used to refine structure: *SHELXL97* (Sheldrick, 2008 ▶); molecular graphics: *PLATON* (Spek, 2009 ▶); software used to prepare material for publication: *PLATON*.

## Supplementary Material

Crystal structure: contains datablocks I, global. DOI: 10.1107/S1600536809047540/im2158sup1.cif

Structure factors: contains datablocks I. DOI: 10.1107/S1600536809047540/im2158Isup2.hkl

Additional supplementary materials:  crystallographic information; 3D view; checkCIF report

## Figures and Tables

**Table 1:** Hydrogen-bond geometry (Å, °)

*D*—H⋯*A*	*D*—H	H⋯*A*	*D*⋯*A*	*D*—H⋯*A*
N1—H1⋯N7^i^	0.95	2.12	3.061 (2)	171

Symmetry code: (i) 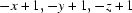.

**Fig. 1.**
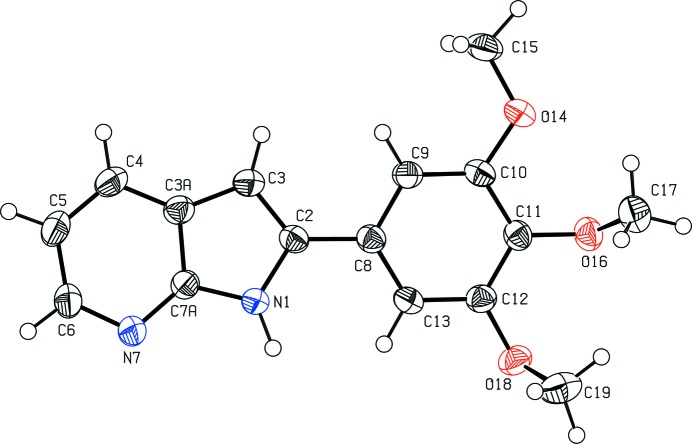
View of compound I. Displacement ellipsoids are drawn at the 50% probability level.

## supplementary crystallographic information

### Comment

7-Azaindoles are found in natural and synthetic compounds of biological interest. The interest in 7-azaindoles as indole analogues has arisen in recent years due to their improved physicochemical and pharmacological properties. The substitution of this heterocycle is widely studied and used in synthesis of many compounds of potential pharmaceutical interest. The 3,4,5-trimethoxyphenyl moiety encloses a dihedral angle of 10.04 (7)° toward the 1*H*-pyrrolo[2,3-*b*]pyridine system. The crystal structure of 2-(3,4,5-trimethoxyphenyl)-1*H*-pyrrolo[2,3-*b*]pyridine, C_16_H_16_N_2_O_3_, is characterized by an intermolecular hydrogen bond N1–H1···N7 (2.12 Å).

### Experimental

3-Methylpyridine (0.68 g 7.25 mmol) was added dropwise to a freshly prepared solution of LDA in THF (2*M*) (3.6 ml 7.25 mmol) at 273 K. The resulting suspension was stirred at 273 K for 30 min. Trimethoxybenzonitrile (1.4 g 7.25 mmol) was added dropwise at such a rate that the temperature did not rise above 283 K. Stirring was continued for 60 min. at 273 K. Another portion of LDA solution (3.6 ml 7.25 mmol) was added and stirring was continued for 10 h at 353 K. The final reaction mixture was allowed to cool and ice-water was added. The mixture was extracted with ethylacetate and the combined extracts were dried (Na_2_SO_4_) and the solvent was evaporated under reduced pressure. The residue was subjected to flash chromatography. The title compound was obtained in a yield of 67% (1.37 g 4.83 mmol). Crystals suitable for X-ray diffraction were obtained by slow evaporation of the solvent (methanol) during several weeks.

### Refinement

Hydrogen atoms attached to carbon were placed at calculated positions with C—H = 0.95 Å (aromatic) or 0.98–0.99 Å (*sp*^3^ C-atom). The H atom attached to N1 was located in difference Fourier maps. All H atoms were refined using the riding-model approximation with isotropic displacement parameters (set at 1.2–1.5 times of the *U*_eq_ of the parent atom).

### Crystal data

**Table:**

C_16_H_16_N_2_O_3_	*F*(000) = 600
*M**_r_* = 284.31	*D*_x_ = 1.353 Mg m^−^^3^
Monoclinic, *P*2_1_/*c*	Cu *K*α radiation, λ = 1.54178 Å
Hall symbol: -P 2ybc	Cell parameters from 25 reflections
*a* = 7.6283 (9) Å	θ = 66–70°
*b* = 10.1745 (4) Å	µ = 0.78 mm^−^^1^
*c* = 18.604 (2) Å	*T* = 193 K
β = 104.778 (6)°	Plate, colourless
*V* = 1396.2 (2) Å^3^	0.40 × 0.40 × 0.25 mm
*Z* = 4	

### Data collection

**Table:**

Enraf–Nonius CAD-4 diffractometer	*R*_int_ = 0.020
Radiation source: rotating anode	θ_max_ = 70.0°, θ_min_ = 4.9°
graphite	*h* = −9→0
ω/2θ scans	*k* = 0→12
2865 measured reflections	*l* = −21→22
2657 independent reflections	3 standard reflections every 60 min
2352 reflections with *I* > 2σ(*I*)	intensity decay: 2%

### Refinement

**Table:**

Refinement on *F*^2^	Primary atom site location: structure-invariant direct methods
Least-squares matrix: full	Secondary atom site location: difference Fourier map
*R*[*F*^2^ > 2σ(*F*^2^)] = 0.045	Hydrogen site location: inferred from neighbouring sites
*wR*(*F*^2^) = 0.125	H-atom parameters constrained
*S* = 1.07	*w* = 1/[σ^2^(*F*_o_^2^) + (0.0641*P*)^2^ + 0.5259*P*] where *P* = (*F*_o_^2^ + 2*F*_c_^2^)/3
2657 reflections	(Δ/σ)_max_ < 0.001
193 parameters	Δρ_max_ = 0.20 e Å^−^^3^
0 restraints	Δρ_min_ = −0.20 e Å^−^^3^

### Special details

**Table:**

Geometry. All e.s.d.'s (except the e.s.d. in the dihedral angle between two l.s. planes) are estimated using the full covariance matrix. The cell e.s.d.'s are taken into account individually in the estimation of e.s.d.'s in distances, angles and torsion angles; correlations between e.s.d.'s in cell parameters are only used when they are defined by crystal symmetry. An approximate (isotropic) treatment of cell e.s.d.'s is used for estimating e.s.d.'s involving l.s. planes.
Refinement. Refinement of *F*^2^ against ALL reflections. The weighted *R*-factor *wR* and goodness of fit *S* are based on *F*^2^, conventional *R*-factors *R* are based on *F*, with *F* set to zero for negative *F*^2^. The threshold expression of *F*^2^ > σ(*F*^2^) is used only for calculating *R*-factors(gt) *etc*. and is not relevant to the choice of reflections for refinement. *R*-factors based on *F*^2^ are statistically about twice as large as those based on *F*, and *R*- factors based on ALL data will be even larger.

### Fractional atomic coordinates and isotropic or equivalent isotropic displacement parameters (Å^2^)

**Table:**

	*x*	*y*	*z*	*U*_iso_*/*U*_eq_	
N1	0.62152 (18)	0.53967 (13)	0.42466 (7)	0.0312 (3)	
H1	0.6399	0.4802	0.4653	0.037*	
C2	0.7269 (2)	0.55266 (16)	0.37403 (8)	0.0308 (3)	
C3	0.6670 (2)	0.65831 (17)	0.32906 (9)	0.0360 (4)	
H3	0.7158	0.6888	0.2899	0.043*	
C3A	0.5187 (2)	0.71430 (17)	0.35131 (9)	0.0344 (4)	
C4	0.4017 (3)	0.82022 (19)	0.32915 (10)	0.0451 (4)	
H4	0.4120	0.8762	0.2896	0.054*	
C5	0.2705 (3)	0.8406 (2)	0.36686 (11)	0.0524 (5)	
H5	0.1888	0.9123	0.3536	0.063*	
C6	0.2568 (3)	0.7568 (2)	0.42413 (11)	0.0492 (5)	
H6	0.1629	0.7736	0.4481	0.059*	
N7	0.3663 (2)	0.65397 (15)	0.44823 (8)	0.0381 (4)	
C7A	0.4931 (2)	0.63661 (16)	0.41105 (9)	0.0310 (3)	
C8	0.8710 (2)	0.45896 (16)	0.37109 (8)	0.0307 (3)	
C9	0.9838 (2)	0.48432 (16)	0.32401 (9)	0.0330 (4)	
H9	0.9711	0.5640	0.2966	0.040*	
C10	1.1137 (2)	0.39363 (17)	0.31741 (9)	0.0324 (4)	
C11	1.1358 (2)	0.27617 (16)	0.35828 (9)	0.0313 (3)	
C12	1.0243 (2)	0.25251 (16)	0.40535 (9)	0.0326 (4)	
C13	0.8925 (2)	0.34277 (16)	0.41142 (9)	0.0338 (4)	
H13	0.8164	0.3249	0.4435	0.041*	
O14	1.22631 (17)	0.40749 (13)	0.27123 (7)	0.0439 (3)	
C15	1.1984 (3)	0.5172 (2)	0.22231 (10)	0.0454 (5)	
H15A	1.0720	0.5186	0.1929	0.068*	
H15B	1.2787	0.5103	0.1889	0.068*	
H15C	1.2253	0.5984	0.2513	0.068*	
O16	1.25139 (16)	0.17927 (12)	0.34718 (7)	0.0376 (3)	
C17	1.4407 (2)	0.2090 (2)	0.37068 (11)	0.0458 (4)	
H17A	1.4722	0.2711	0.3357	0.069*	
H17B	1.5110	0.1281	0.3721	0.069*	
H17C	1.4684	0.2484	0.4204	0.069*	
O18	1.03203 (16)	0.13515 (12)	0.44277 (7)	0.0399 (3)	
C19	1.1896 (3)	0.1164 (2)	0.50170 (11)	0.0518 (5)	
H19A	1.2953	0.1062	0.4813	0.078*	
H19B	1.1753	0.0372	0.5297	0.078*	
H19C	1.2073	0.1928	0.5349	0.078*	

### Atomic displacement parameters (Å^2^)

**Table:**

	*U*^11^	*U*^22^	*U*^33^	*U*^12^	*U*^13^	*U*^23^
N1	0.0357 (7)	0.0303 (7)	0.0307 (7)	0.0044 (5)	0.0140 (5)	0.0038 (5)
C2	0.0334 (8)	0.0312 (8)	0.0295 (7)	−0.0014 (6)	0.0112 (6)	−0.0015 (6)
C3	0.0419 (9)	0.0371 (9)	0.0310 (8)	0.0024 (7)	0.0134 (7)	0.0049 (7)
C3A	0.0397 (9)	0.0348 (8)	0.0283 (8)	0.0025 (7)	0.0082 (6)	0.0019 (6)
C4	0.0566 (11)	0.0429 (10)	0.0369 (9)	0.0136 (9)	0.0135 (8)	0.0129 (8)
C5	0.0596 (12)	0.0519 (12)	0.0470 (10)	0.0276 (10)	0.0161 (9)	0.0138 (9)
C6	0.0513 (11)	0.0563 (12)	0.0445 (10)	0.0241 (9)	0.0203 (8)	0.0092 (9)
N7	0.0427 (8)	0.0400 (8)	0.0345 (7)	0.0116 (6)	0.0149 (6)	0.0044 (6)
C7A	0.0346 (8)	0.0298 (8)	0.0281 (7)	0.0025 (6)	0.0072 (6)	−0.0008 (6)
C8	0.0328 (8)	0.0304 (8)	0.0301 (7)	−0.0012 (6)	0.0101 (6)	−0.0018 (6)
C9	0.0383 (8)	0.0310 (8)	0.0317 (8)	−0.0001 (7)	0.0127 (7)	0.0024 (6)
C10	0.0358 (8)	0.0356 (9)	0.0289 (7)	−0.0028 (7)	0.0138 (6)	−0.0018 (6)
C11	0.0334 (8)	0.0300 (8)	0.0318 (8)	0.0003 (6)	0.0104 (6)	−0.0045 (6)
C12	0.0361 (8)	0.0287 (8)	0.0339 (8)	−0.0024 (6)	0.0109 (6)	0.0016 (6)
C13	0.0347 (8)	0.0360 (9)	0.0346 (8)	−0.0002 (7)	0.0162 (7)	0.0027 (7)
O14	0.0502 (7)	0.0475 (8)	0.0431 (7)	0.0089 (6)	0.0285 (6)	0.0092 (6)
C15	0.0417 (9)	0.0565 (12)	0.0424 (10)	−0.0042 (8)	0.0184 (8)	0.0123 (8)
O16	0.0388 (6)	0.0343 (6)	0.0422 (6)	0.0041 (5)	0.0148 (5)	−0.0042 (5)
C17	0.0393 (10)	0.0520 (11)	0.0466 (10)	0.0033 (8)	0.0121 (8)	−0.0037 (9)
O18	0.0414 (7)	0.0327 (6)	0.0457 (7)	−0.0015 (5)	0.0113 (5)	0.0095 (5)
C19	0.0540 (12)	0.0544 (12)	0.0426 (10)	−0.0083 (9)	0.0041 (9)	0.0139 (9)

### Geometric parameters (Å, °)

**Table:**

N1—C7A	1.367 (2)	C10—O14	1.3681 (19)
N1—C2	1.3917 (19)	C10—C11	1.403 (2)
N1—H1	0.9499	C11—O16	1.3729 (19)
C2—C3	1.367 (2)	C11—C12	1.389 (2)
C2—C8	1.467 (2)	C12—O18	1.3758 (19)
C3—C3A	1.419 (2)	C12—C13	1.387 (2)
C3—H3	0.9500	C13—H13	0.9500
C3A—C4	1.393 (2)	O14—C15	1.422 (2)
C3A—C7A	1.417 (2)	C15—H15A	0.9800
C4—C5	1.377 (3)	C15—H15B	0.9800
C4—H4	0.9500	C15—H15C	0.9800
C5—C6	1.389 (3)	O16—C17	1.430 (2)
C5—H5	0.9500	C17—H17A	0.9800
C6—N7	1.343 (2)	C17—H17B	0.9800
C6—H6	0.9500	C17—H17C	0.9800
N7—C7A	1.336 (2)	O18—C19	1.418 (2)
C8—C13	1.387 (2)	C19—H19A	0.9800
C8—C9	1.400 (2)	C19—H19B	0.9800
C9—C10	1.382 (2)	C19—H19C	0.9800
C9—H9	0.9500		
			
C7A—N1—C2	108.43 (13)	O14—C10—C11	114.85 (14)
C7A—N1—H1	124.1	C9—C10—C11	120.66 (14)
C2—N1—H1	127.2	O16—C11—C12	119.38 (15)
C3—C2—N1	109.17 (14)	O16—C11—C10	121.53 (14)
C3—C2—C8	128.68 (14)	C12—C11—C10	118.76 (14)
N1—C2—C8	122.09 (14)	O18—C12—C13	118.16 (14)
C2—C3—C3A	107.69 (14)	O18—C12—C11	121.02 (14)
C2—C3—H3	126.2	C13—C12—C11	120.64 (15)
C3A—C3—H3	126.2	C12—C13—C8	120.57 (14)
C4—C3A—C7A	117.21 (16)	C12—C13—H13	119.7
C4—C3A—C3	136.27 (16)	C8—C13—H13	119.7
C7A—C3A—C3	106.52 (14)	C10—O14—C15	117.87 (13)
C5—C4—C3A	117.38 (16)	O14—C15—H15A	109.5
C5—C4—H4	121.3	O14—C15—H15B	109.5
C3A—C4—H4	121.3	H15A—C15—H15B	109.5
C4—C5—C6	120.36 (17)	O14—C15—H15C	109.5
C4—C5—H5	119.8	H15A—C15—H15C	109.5
C6—C5—H5	119.8	H15B—C15—H15C	109.5
N7—C6—C5	124.90 (17)	C11—O16—C17	116.03 (13)
N7—C6—H6	117.5	O16—C17—H17A	109.5
C5—C6—H6	117.6	O16—C17—H17B	109.5
C7A—N7—C6	113.64 (15)	H17A—C17—H17B	109.5
N7—C7A—N1	125.32 (14)	O16—C17—H17C	109.5
N7—C7A—C3A	126.50 (15)	H17A—C17—H17C	109.5
N1—C7A—C3A	108.18 (14)	H17B—C17—H17C	109.5
C13—C8—C9	119.24 (15)	C12—O18—C19	115.25 (14)
C13—C8—C2	121.36 (14)	O18—C19—H19A	109.5
C9—C8—C2	119.33 (14)	O18—C19—H19B	109.5
C10—C9—C8	120.12 (15)	H19A—C19—H19B	109.5
C10—C9—H9	119.9	O18—C19—H19C	109.5
C8—C9—H9	119.9	H19A—C19—H19C	109.5
O14—C10—C9	124.47 (15)	H19B—C19—H19C	109.5
			
C7A—N1—C2—C3	0.71 (18)	C13—C8—C9—C10	0.7 (2)
C7A—N1—C2—C8	−176.55 (14)	C2—C8—C9—C10	−176.40 (14)
N1—C2—C3—C3A	−0.21 (19)	C8—C9—C10—O14	177.84 (15)
C8—C2—C3—C3A	176.82 (16)	C8—C9—C10—C11	−0.8 (2)
C2—C3—C3A—C4	179.8 (2)	O14—C10—C11—O16	−5.2 (2)
C2—C3—C3A—C7A	−0.35 (19)	C9—C10—C11—O16	173.52 (14)
C7A—C3A—C4—C5	−0.2 (3)	O14—C10—C11—C12	−178.56 (14)
C3—C3A—C4—C5	179.6 (2)	C9—C10—C11—C12	0.2 (2)
C3A—C4—C5—C6	−0.6 (3)	O16—C11—C12—O18	2.1 (2)
C4—C5—C6—N7	1.1 (4)	C10—C11—C12—O18	175.55 (14)
C5—C6—N7—C7A	−0.7 (3)	O16—C11—C12—C13	−172.96 (15)
C6—N7—C7A—N1	179.77 (17)	C10—C11—C12—C13	0.5 (2)
C6—N7—C7A—C3A	−0.1 (3)	O18—C12—C13—C8	−175.80 (14)
C2—N1—C7A—N7	179.17 (15)	C11—C12—C13—C8	−0.6 (2)
C2—N1—C7A—C3A	−0.92 (18)	C9—C8—C13—C12	0.0 (2)
C4—C3A—C7A—N7	0.6 (3)	C2—C8—C13—C12	177.04 (15)
C3—C3A—C7A—N7	−179.31 (16)	C9—C10—O14—C15	−5.3 (2)
C4—C3A—C7A—N1	−179.33 (15)	C11—C10—O14—C15	173.38 (15)
C3—C3A—C7A—N1	0.79 (18)	C12—C11—O16—C17	−117.52 (17)
C3—C2—C8—C13	−167.47 (17)	C10—C11—O16—C17	69.2 (2)
N1—C2—C8—C13	9.2 (2)	C13—C12—O18—C19	−113.18 (18)
C3—C2—C8—C9	9.5 (3)	C11—C12—O18—C19	71.7 (2)
N1—C2—C8—C9	−173.77 (14)		

### Hydrogen-bond geometry (Å, °)

**Table:**

*D*—H···*A*	*D*—H	H···*A*	*D*···*A*	*D*—H···*A*
N1—H1···N7^i^	0.95	2.12	3.061 (2)	171

Symmetry codes: (i) −*x*+1, −*y*+1, −*z*+1.
